# Temporal Reduction in COVID-19-Associated Fatality Among Kidney Transplant Recipients: The Brazilian COVID-19 Registry Cohort Study

**DOI:** 10.3389/ti.2022.10205

**Published:** 2022-02-01

**Authors:** Tainá Veras de Sandes-Freitas, Marina Pontello Cristelli, Lucio Roberto Requião-Moura, Luís Gustavo Modelli de Andrade, Laila Almeida Viana, Valter Duro Garcia, Claudia Maria Costa de Oliveira, Ronaldo de Matos Esmeraldo, Paula Roberta de Lima, Ida Maria Maximina Fernandes Charpiot, Teresa Cristina Alves Ferreira, Rodrigo Fontanive Franco, Kellen Micheline Alves Henrique Costa, Denise Rodrigues Simão, Gustavo Fernandes Ferreira, Viviane Brandão Bandeira de Mello Santana, Ricardo Augusto Monteiro de Barros Almeida, Luciane Monica Deboni, Anita Leme da Rocha Saldanha, Irene de Lourdes Noronha, Lívia Cláudio de Oliveira, Deise De Boni Monteiro de Carvalho, Reinaldo Barreto Oriá, Jose Osmar Medina-Pestana, Helio Tedesco-Silva Junior

**Affiliations:** ^1^ Programa de Pós-Graduação em Ciências Médicas, Departamento de Medicina Clínica, Faculdade de Medicina, Universidade Federal do Ceará, Fortaleza, Brazil; ^2^ Hospital Universitário Walter Cantídio, Fortaleza, Brazil; ^3^ Hospital Geral de Fortaleza, Fortaleza, Brazil; ^4^ Hospital do Rim, Fundção Oswaldo Ramos, São Paulo, Brazil; ^5^ Departamento de Medicina, Divisão de Nefrologia, Universidade Federal de São Paulo, São Paulo, Brazil; ^6^ Unidade de Transplante Renal, Hospital Israelita Albert Einstein, São Paulo, Brazil; ^7^ Departamento de Medicina Interna, Universidade Estadual Paulista-UNESP, Botucatu, Brazil; ^8^ Santa Casa de Misericórdia de Porto Alegre, Porto Alegre, Brazil; ^9^ Hospital de Base, Faculdade de Medicina de São José do Rio Preto (FAMERP), São José do Rio Preto, Brazil; ^10^ Universidade Federal do Maranhão, São Luiz, Brazil; ^11^ Hospital de Clínicas de Porto Alegre, Universidade Federal do Rio Grande do Sul, Porto Alegre, Brazil; ^12^ Divisão de Nefrologia e Transplante Renal, Hospital Universitário Onofre Lopes (HOUL), Natal, Brazil; ^13^ Hospital Santa Isabel, Blumenau, Brazil; ^14^ Santa Casa de Misericórdia de Juiz de Fora, Juiz de Fora, Brazil; ^15^ Hospital de Base do Distrito Federal, Brasilia, Brazil; ^16^ Hospital Municipal São José (HMSJ), Joinville, Brazil; ^17^ Hospital Beneficência Portuguesa de São Paulo (BP), São Paulo, Brazil; ^18^ Divisão de Nefrologia, Hospital das Clínicas, Faculdade de Medicina, Universidade de São Paulo, São Paulo, Brazil; ^19^ Unidade de Transplantes, Hospital Universitário de Brasília, Universidade de Brasília (UnB), Brasília, Brazil; ^20^ Hospital São Francisco na Providência de Deus, Rio de Janeiro, Brazil

**Keywords:** Sars-CoV-2, Covid-19, kidney transplant, coronavirus, renal transplantation

## Abstract

Data from the general population suggest that fatality rates declined during the course of the pandemic. This analysis, using data extracted from the Brazilian Kidney Transplant COVID-19 Registry, seeks to determine fatality rates over time since the index case on March 3rd, 2020. Data from hospitalized patients with RT-PCR positive SARS-CoV-2 infection from March to August 2020 (35 sites, 878 patients) were compared using trend tests according to quartiles (Q1: <72 days; Q2: 72–104 days; Q3: 105–140 days; Q4: >140 days after the index case). The 28-day fatality decreased from 29.5% (Q1) to 18.8% (Q4) (*p*_*for-trend*_ = 0.004). In multivariable analysis, patients diagnosed in Q4 showed a 35% reduced risk of death. The trend of reducing fatality was associated with a lower number of comorbidities (20.7–10.6%, p_*for-trend*_ = 0.002), younger age (55–53 years, *p*_*for-trend*_ = 0.062), and better baseline renal function (43.6–47.7 ml/min/1.73 m^2^, *p*_*for-trend*_ = 0.060), and were confirmed by multivariable analysis. The proportion of patients presenting dyspnea (*p*_*for-trend*_ = 0.001) and hypoxemia (*p*_*for-trend*_ < 0.001) at diagnosis, and requiring intensive care was also found reduced (*p*_*for-trend*_ = 0.038). Despite possible confounding variables and time-dependent sampling differences, we conclude that COVID-19-associated fatality decreased over time. Differences in demographics, clinical presentation, and treatment options might be involved.

## Graphical Abstract

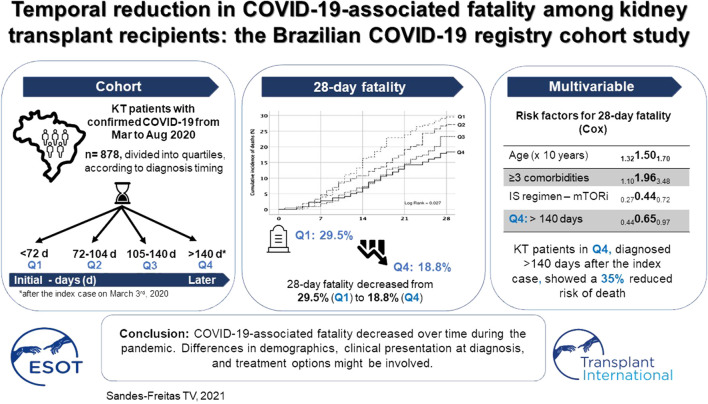

## Introduction

Over the past year, the coronavirus disease 2019 (COVID-19) global pandemic has been responsible for more than 126 million cases of severe acute respiratory syndrome worldwide and over 2.76 million deaths. With large numbers of COVID cases, Brazil has become an epicenter of the COVID-19 outbreak in the world (1, 2). Among many specific vulnerable groups affected by SARS-COV-2 infection, transplant immunocompromised recipients represent a recognized high-risk group for this infection (3).

Although to date there is still no specific treatment for COVID-19, several pharmacological and non-pharmacological strategies have been explored to improve the clinical outcomes. Among these strategies, the following are noteworthy: 1) the use of prehospital pulse oximetry to early detect silent hypoxemia (4); 2) the important role of non-invasive mechanical ventilation often avoiding unnecessary early intubation (5); 3) prone position to improve oxygenation in intubated and non-intubated patients with COVID-19-related acute respiratory distress syndrome (6, 7); 4) anticoagulant treatment in patients with coagulopathy (8); and 5) corticosteroids in patients with severe disease (9).

Data from the general population suggest an improvement in survival rates during the pandemic, mainly among critically ill patients (10–13). Multicenter national studies have reported COVID-19-related fatality rates varying from 20.5 to 32% among hospitalized kidney transplant (KT) patients (14–18), but no study evaluated the impact of the timing on deaths in this population.

In this analysis of the multicenter national Brazilian registry of SARS-CoV-2 infection study, we aimed to assess fatality rates over the first 6 months of pandemic and to explore whether demographics, clinical profile, and in-hospital management of COVID-19 were associated with trends in the outcomes.

## Materials and Methods

### Study Design

This is an ongoing multicenter national Brazilian registry of SARS-CoV-2 infection among kidney transplant recipients (ClinicalTrials.gov: NCT04494776) (19). For this analysis, we extracted data of patients with COVID-19-related signs and symptoms and SARS-CoV-2 detected by reverse-transcription polymerase chain reaction (RT-PCR) of a respiratory sample, between 3rd March and 31st August 2020, who required hospitalization, totalizing 878 patients from 35 transplant centers of four Brazilian Regions (615 from the Southeast, 124 Northeast, 111 South, and 28 from the Midwest). Patients were followed for 3 months after the diagnosis or until death or graft loss, and the end-of-study data was 30th November 2020.

### Variables

Patient age, gender, ethnicity, and body mass index were collected and included in the analysis. Comorbidities comprised the following conditions: hypertension, diabetes, cardiovascular, pulmonary, neurological or hepatic diseases, current or previous neoplasia, and autoimmune disease. The following clinical presentation parameters were also included in the analysis: fever and/or chills, cough, dyspnea, myalgia, diarrhea, headache, fatigue and or/asthenia, runny nose, and nausea and/or vomiting. Data related to KT such as donor source, end-stage kidney disease (ESKD) etiology, time after transplantation, baseline renal function, maintenance immunosuppressive (IS) drugs, steroid (ST) pulse therapy <3 months, use of rabbit antithymocyte globulin (rATG) <3 months were analyzed.

The following laboratory exams at admission were recorded: lymphocytes count, hemoglobin, platelets count, C-reactive protein, lactic dehydrogenase, aspartate transaminase; alanine transaminase; creatine phosphokinase, serum sodium, ferritin, serum creatinine. Chest radiography and/or computed tomography at admission were used to classify pulmonary abnormalities.

The following treatments available in the registry were analyzed: antibiotics, particularly azithromycin, high-dose steroids, prophylactic or therapeutic use of anticoagulants, and use of oseltamivir, ivermectin, and chloroquine or hydroxychloroquine.

The analysis of outcomes in COVID-19 transplant recipients across time was carried out considering fatality rates and the following variables: invasive mechanical ventilation, intensive care unit admission, and development of AKI with dialysis requirement.

### Definitions

The COVID-19-associated fatality rate was defined as the percentage of deaths that occurred in patients with confirmed SARS-CoV-2 infection. Hospital admission criteria and the use of pharmacological and non-pharmacological treatments were at the discretion of each of the participating centers. The definition of “high-dose steroids” was at the center discretion, according to their local practices.

**FIGURE 1:**
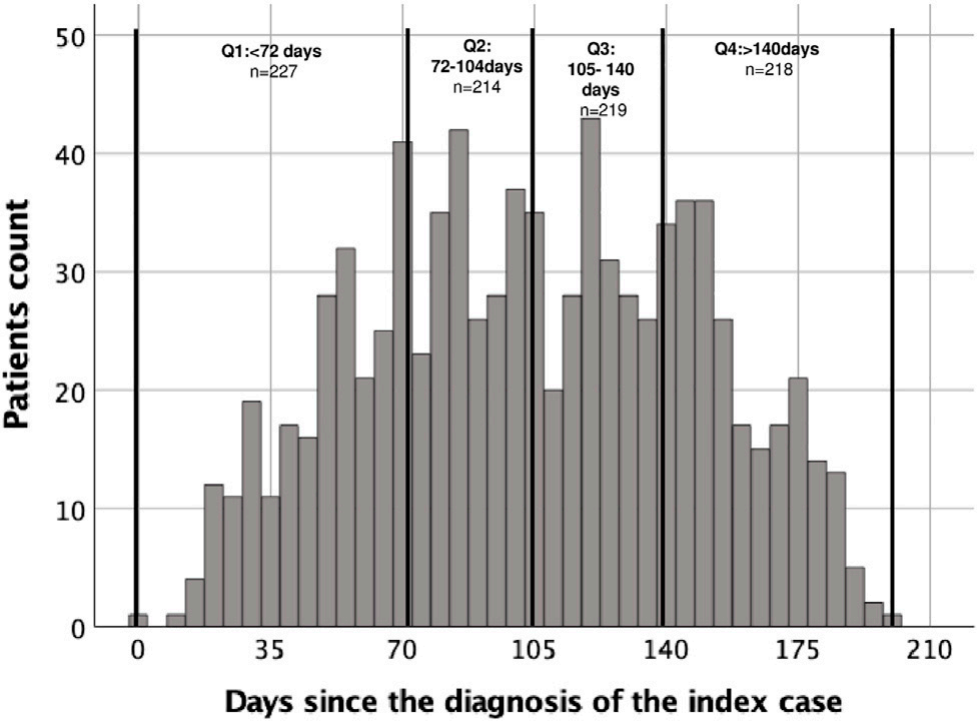
Distribution of COVID-19 diagnosed transplant patients after the index case, on March 3rd, 2020, according to quartiles.

We considered as the index case the first KT patient diagnosed with COVID-19 and included in the Brazilian Kidney Transplant COVID-19 Registry (March 3rd, 2020). The sample was divided into quartiles, as demonstrated in Figure 1: Q1: patients diagnosed <72 days after the index case (*n* = 227); Q2: 72–104 days (*n* = 214); Q3: 105–140 days (*n* = 219); Q4: >140 days (*n* = 218).

Baseline serum creatinine (sCr) was defined as the last three available sCr measurements before COVID-19 infection. Glomerular filtration rate (eGFR) was estimated by the CKD-EPI formula. Delta sCr (Δ sCr) was the difference between admission and baseline sCr values. Acute kidney injury (AKI) was defined as a rise in sCr of ≥50% from its baseline value (20). Graft loss was defined as the return to long-term dialysis therapy or retransplantation.

### Statistical Analysis

Categorical variables were presented as frequency and percentage. All continuous variables were non-normally distributed and were summarized as median and interquartile range (IQR). Trend analyses comparing data across the quartiles were performed using Cochran–Armitage test for categorical variables, and Jonckheere-Terpstra test for numerical variables. Survival curves were obtained using Kaplan-Meier method and compared using the log-rank test. Univariable and multivariable analyses to identify independent risk factors associated with death were performed using Cox regression, with center-based random effects (frailty model). Collinear variables, and those poorly associated with death in univariable analysis (*p* > 0.15) were excluded from the multivariable model. No variable exceeded 5% of missing values and Multiple Imputation by Chained Equation (MICE) was used to replace missing data values, as follows: 1) generating replacement values (“imputations”) for missing data and repeating this procedure 10 times, 2) analyzing the 10 imputed data sets, and 3) combining (pooling) the results using Rubin’s Rules (21). A significantly statistical difference was assumed when the *p*-value was less than 0.05. Statistical analysis was performed using the IBM SPSS 25 and R 4.0.2.

## Results

### Demographic Characteristics Across the Quartiles

The baseline demographic characteristics at COVID-19 diagnosis are shown in Table 1. Changes in patients’ clinical profile occurred over time, with a significant reduction in age, and in the percentage of patients with ≥3 comorbidities.

**TABLE 1:** Demographic characteristics of kidney transplanted patients at COVID-19 diagnosis across quartiles of time.

	Non-missing cases	Total	Q1	Q2	Q3	Q4	*p*_*for-trend*_
		*N* = 878	*N* = 227	*N* = 214	*N* = 219	*N* = 218	
Age (years-old)	878	54 (45–62)	55 (46–64)	54 (44–61)	54 (45–61)	53 (44–62)	0.062
Male gender	878	535 (60.9)	146 (64.3)	131 (61.2)	134 (61.2)	124 (56.9)	0.127
Ethnicity	878						0.204
Caucasian		483 (55.0)	111 (48.9)	108 (50.5)	125 (57.1)	139 (63.8)	
Mixed race		255 (29.0)	79 (34.8)	68 (31.8)	63 (28.8)	45 (20.6)	
Afro-Brazilian		112 (12.8)	28 (12.3)	28 (13.1)	24 (11.0)	32 (14.7)	
Asian		14 (1.6)	6 (2.6)	3 (1.4)	4 (1.8)	1 (0.5)	
Indian		1 (0.1)	0 (0)	0 (0)	1 (0.5)	0 (0)	
Not available		13 (1.5)	3 (1.3)	7 (3.3)	2 (0.9)	1 (0.5)	
BMI (kg/m^2^)	842	26.5 (23.6–30.0)	26.4 (23.3–29.5)	26.0 (22.9–29.7)	27.3 (24.4–30.9)	26.8 (23.9–29.9)	**0.031**
Donor source	878						0.084
KT - LD		259 (29.5)	79 (34.8)	62 (29.0)	67 (30.6)	51 (23.4)	
KT - DD		601 (68.5)	142 (62.6)	151 (70.6)	146 (66.7)	162 (74.3)	
Combined KT^a^		18 (2.1)	6 (0.7)	1 (0.1)	6 (0.7)	5 (0.6)	
ESKD etiology	878						0.230
Unknown		266 (30.3)	57 (25.1)	80 (37.4)	69 (31.5)	60 (27.5)	
Diabetes		174 (19.8)	53 (23.3)	41 (19.2)	38 (17.4)	42 (19.3)	
Chronic GN		151 (17.2)	33 (14.5)	30 (14.0)	51 (23.3)	37 (17.0)	
Hypertension		103 (11.7)	34 (15.0)	22 (10.3)	20 (9.1)	27 (12.4)	
PKD		73 (8.3)	20 (8.8)	14 (6.5)	19 (8.7)	20 (9.2)	
Urological		14 (1.6)	4 (1.8)	4 (1.9)	3 (1.4)	3 (1.4)	
Other		97 (11.0)	26 (11.5)	23 (10.7)	19 (8.7)	29 (13.3)	
Time after KT (years)	875	6.1 (2.2–11.2)	6.9 (2.5–11.8)	5.6 (2.1–10.3)	6.1 (2.0–11.7)	5.7 (2.5–11.2)	0.541
Comorbidities	878						
Hypertension		689 (78.5)	179 (78.9)	170 (79.4)	175 (79.9)	165 (75.7)	0.471
Diabetes		351 (40.0)	101 (44.5)	84 (39.3)	89 (40.6)	77 (35.2)	0.075
Cardiovascular disease		142 (16.2)	49 (21.6)	33 (23.2)	32 (14.6)	28 (12.8)	**0.014**
Pulmonary disease		30 (3.4)	10 (4.4)	7 (3.3)	7 (3.2)	6 (2.8)	0.353
Neurological disease		10 (1.1)	5 (2.2)	1 (0.5)	1 (0.5)	3 (1.4)	0.416
Hepatic disease		35 (4.0)	8 (3.5)	8 (3.7)	8 (3.7)	11 (5.0)	0.449
Current or previous neoplasia		59 (6.7)	31 (13.7)	14 (6.5)	10 (4.6)	4 (1.8)	**<0.001**
Autoimmune disease		22 (2.5)	11 (4.8)	2 (0.9)	6 (2.7)	3 (1.4)	0.062
No. of comorbidities	878						**0.002**
None		111 (12.6)	23 (10.1)	26 (12.1)	31 (14.2)	31 (14.2)	
1–2		644 (73.3)	157 (69.2)	161 (75.2)	162 (74.0)	164 (75.2)	
3 or more		123 (14.0)	47 (20.7)	27 (12.6)	26 (11.9)	23 (10.6)	
Maintenance IS drugs	872						
CNI		691 (79.2)	170 (74.9)	170 (79.8)	180 (83.3)	171 (79.2)	0.172
MPA or AZA		653 (74.9)	163 (71.8)	152 (71.4)	167 (77.3)	171 (79.2)	**0.033**
mTORi		135 (15.5)	40 (17.9)	42 (19.7)	26 (12.2)	267 (12.7)	**0.038**
ST		826 (94.7)	212 (93.4)	203 (94.9)	202 (92.2)	209 (95.9)	0.496
RAAS blockade	866	294 (33.9)	74 (32.6)	65 (30.4)	76 (34.7)	79 (36.2)	0.787
ST pulse therapy ≤3 months	859	49 (5.7)	11 (4.8)	7 (3.3)	12 (5.5)	19 (8.7)	0.460
rATG ≤3 months	844	30 (3.6)	8 (3.5)	6 (2.8)	7 (3.2)	9 (4.1)	0.222
eGFR (ml/min/1.73 m^2^)	846	44.5 (28.7–60.9)	43.6 (25.4–57.9)	46.3 (30.0–61.1)	40.9 (27.3–59.3)	47.7 (31.9–66.7)	0.060

Trend analysis for categorical and continuous data were performed using Cochran–Armitage test and Jonckheere-Terpstra test, respectively. BMI, body mass index; KT, kidney transplant; LD, living donor; DD, deceased donor; CNI, calcineurin inhibitor; AZA, azathioprine; MPA, mycophenolate; mTORi, mammalian target of rapamycin inhibitor; RAAS, renin-angiotensin-aldosterone system; ST, steroids; rATG, rabbit antithymocyte globulin; ESKD, end-stage kidney disease; GN, glomerulonephritis; PKD, polycystic kidney disease; IS, immunosuppressive; eGFR, estimated glomerular filtration rate.

Bold values denote statistical significance at the *p* < 0.05 level.

a Simultaneous pancreas-kidney = 8; simultaneous liver-kidney = 6; kidney after liver = 3; simultaneous heart-kidney = 1.

### The Clinical Presentation Across the Quartiles

**FIGURE 2:**
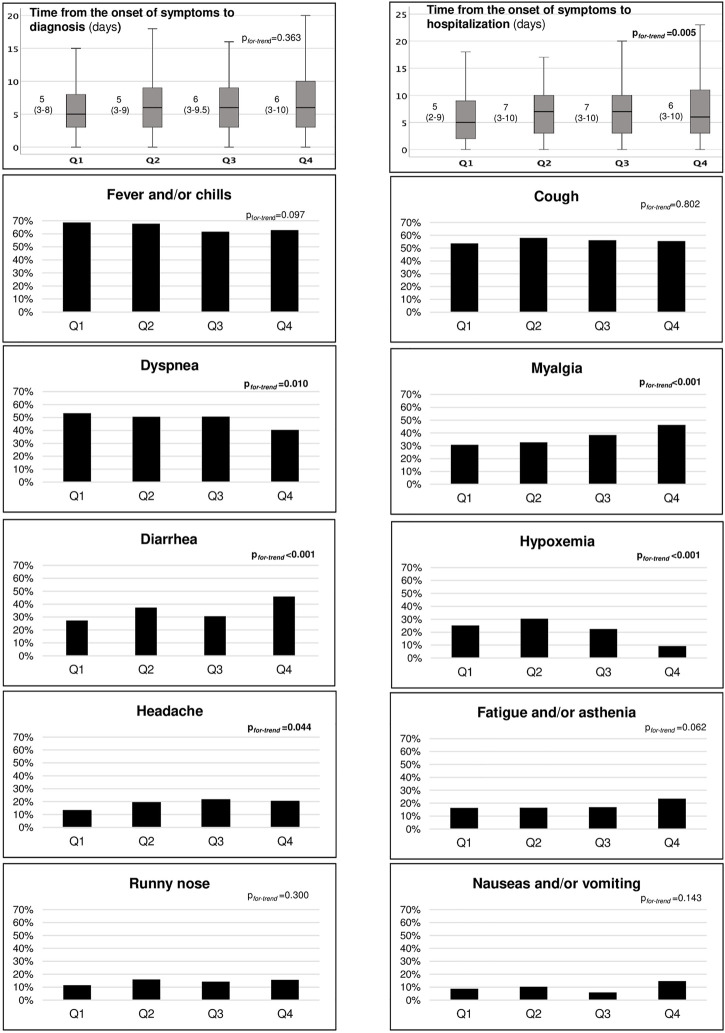
Main signs and symptoms at COVID-19 diagnosis across the quartiles. Trend analyses were performed using Cochran–Armitage test and Jonckheere-Terpstra test.

The analysis across quartiles showed a decrease in the proportion of patients with dyspnea and hypoxemia at diagnosis, whereas myalgia, diarrhea, and headache progressively increased. Although the time from the onset of COVID-19 symptoms to diagnosis remained stable over time (median 6 days; IQR 3–9), a longer time until hospitalization since symptoms onset was observed, increasing from Q1 (median 5 days, IQR 2–9) to Q4 (median 6 days, IQR 3–10) (*p*_*for-trend*_ = 0.005) (Figure 2).

Laboratory data and chest radiological findings at COVID-19 diagnosis are shown in Supplementary Table S1. An increase in the percentage of patients with normal chest radiological evaluation was observed from Q1 (2.1%) to Q4 (6.7%) (*p*_*for-trend*_ = 0.015).

### Immunosuppression and Pharmacological Treatment Across the Quartiles

**FIGURE 3:**
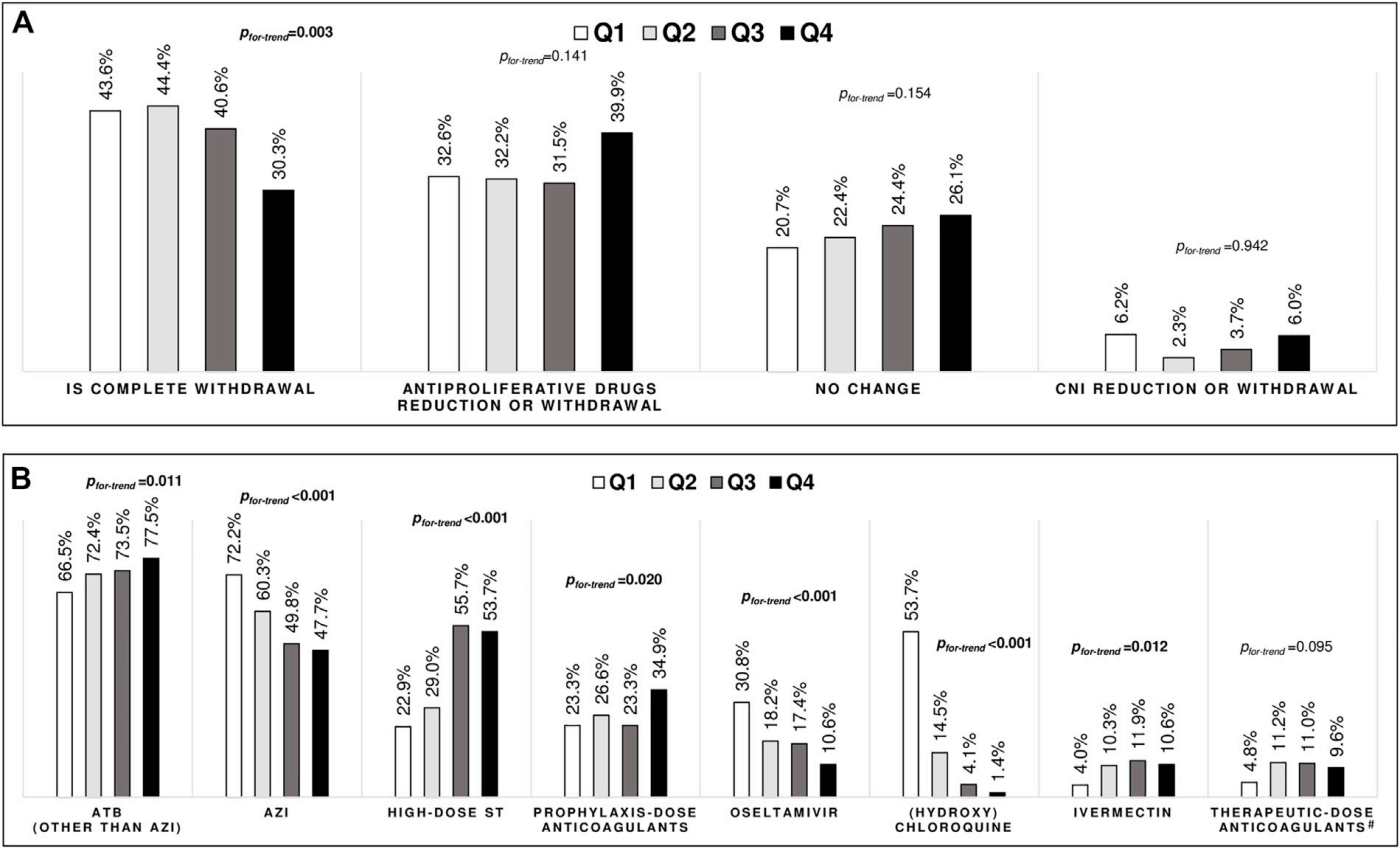
Management of immunosuppressive drugs **(A)** and pharmacological treatments **(B)** across the quartiles. Legend: IS, immunosuppressive drugs; CNI, calcineurin inhibitor; ATB, antibiotics; AZI, azithromycin; ST, steroids. Trend analyses were performed using Cochran–Armitage test ^#^Therapeutic-dose anticoagulants was empirically used for critically il patients with high d-dimer values, regardless of thrombosis events.

Complete immunosuppressive drug withdrawal decreased from Q1 to Q4 (from 43.6 to 30.3%, *p*_*for-trend*_ = 0.003), while no significant changes were observed in the percentage of patients submitted to withdrawal or reduction of the antiproliferative or calcineurin inhibitors agents, or no intervention on the immunosuppressive regimen (Figure 3A).

Regarding the pharmacological treatments, there was an increase in the use of antibiotics, high-dose steroids, prophylactic use of anticoagulants, and ivermectin, while the use of azithromycin, oseltamivir, chloroquine, or hydroxychloroquine decreased from Q1 to Q4 (Figure 3B).

### The Outcomes Across the Quartiles

**FIGURE 4:**
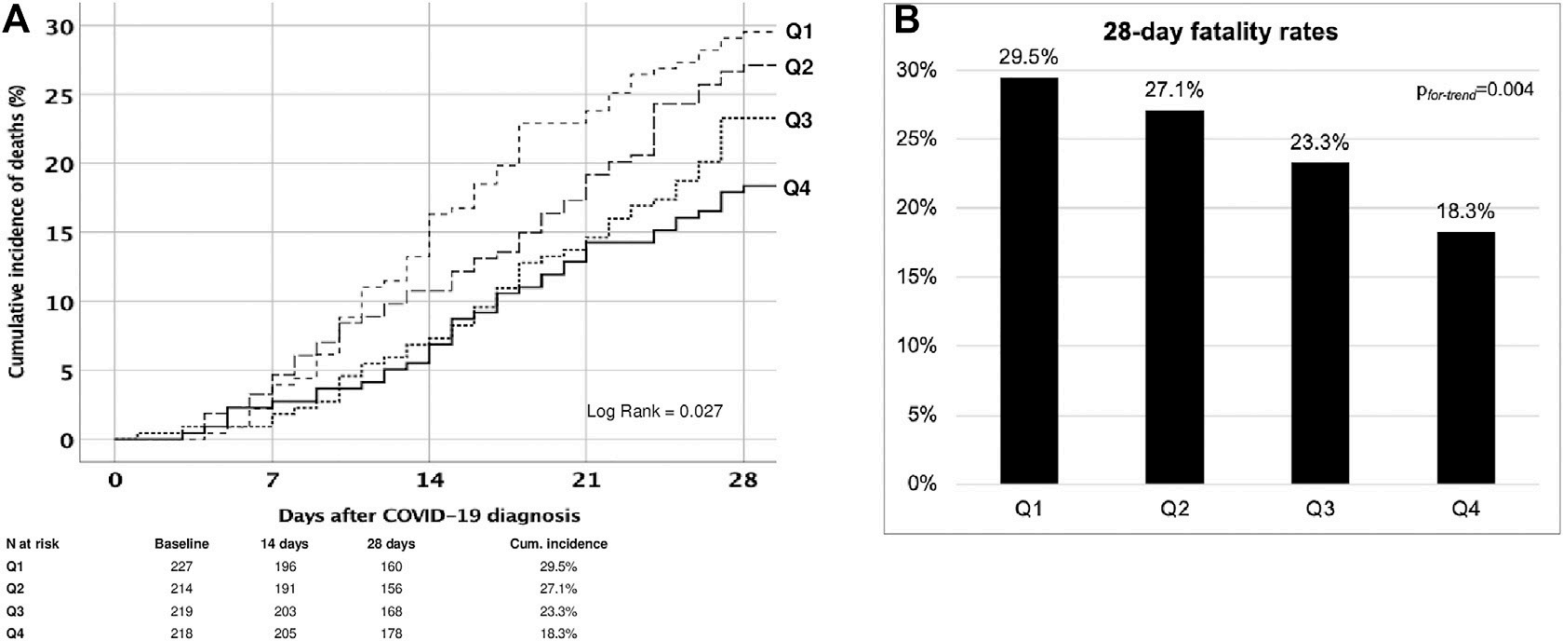
Cumulative incidence of deaths of SARS-CoV-2-infected kidney transplant patients within 28 days. **(A)** and 28-day fatality rates **(B)** across the quartiles.

The 28-day fatality rate was 24.6% (*n* = 216), with a significant downward trend over time, from 29.5% in Q1 to 18.3% in Q4 (log rank = 0.027, *p*_*for-trend*_ = 0.004) (Figures 4A,B).

Causes of death within 28 days included septic shock (60.2%), acute respiratory failure (21.8%), cardiovascular or embolic event (5.1%), and in 13% the cause of death was not clearly defined nor registered. No difference in the distribution of the causes of death occurred from Q1 to Q4 (*p*_*for-trend*_ = 0.677). Although 69.5% of deaths occurred in the first 28 days, the median time from COVID-19 diagnosis to death increased from 17 days (Q1) to 25 days (Q4) (*p*_*for-trend*_ = 0.035). Within the 90-day follow-up, the overall fatality rate was 35.4% (*n* = 311), with a non-significant downward trend from 39.2 to 31.2% (Log-rank = 0.208, *p*_*for-trend*_ = 0.073) (Supplementary Figure S1). Causes of death within 90 days were similar to that described for 28 days.

**FIGURE 5:**
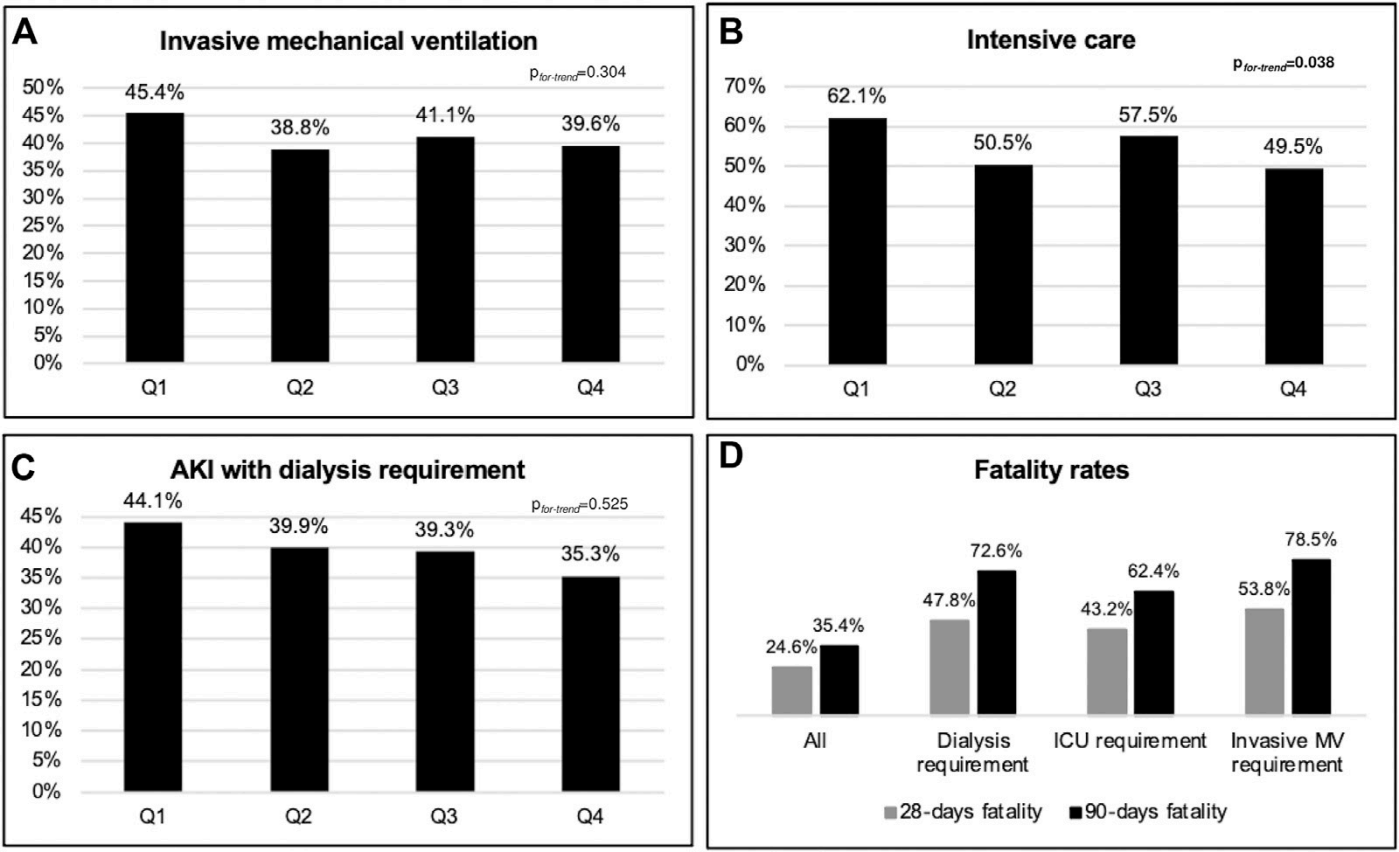
Outcomes after SARS-CoV-2 infection in kidney transplant patients across the quartiles **(A–C)** and fatality rates **(D)**. Legend: AKI, acute kidney injury; ICU, intensive care unit; MV, mechanical ventilation. Trend analyses were performed using Cochran–Armitage test.

No changes were observed in the percentage of patients receiving invasive mechanical ventilation. However, the time from the onset of symptoms to orotracheal intubation increased from 8 to 11 days in median (*p*_*for-trend*_ = <0.001), and fewer patients were admitted to intensive care units (ICU) over time (from 62.1 to 49.5%, *p*_*for-trend*_ = 0.038) (Figures 5A,B). No significant trend was observed in the percentage of patients requiring dialysis therapy (Figure 5C).

Fourteen (1.6%) patients lost the graft within the 90 days follow-up, most of them with advanced chronic kidney disease at the time of COVID-19 diagnosis (median baseline eGFR 16.9 ml/min/1.73 m^2^, IQR, 9.5–24.3) (Supplementary Table S2). Figure 5D shows the 28 and 90-day fatality rates in patients requiring dialysis therapy, ICU admission, and invasive mechanical ventilation.

Patients with COVID-19 diagnosis 140 days after the index case (Q4) showed a 35% reduction risk in 28-day mortality (HR 0.65, 95% CI 0.44–0.97, *p* = 0.037). Each month after March 3rd was associated with 10% reduction in the fatality (HR 0.90, 95% CI 0.82–0.99), *p* = 0.024). Age and presence of three or more comorbidities in addition to chronic kidney disease were also risk factors associated with increased risk of death, whereas the use of mTOR inhibitor and the increasing baseline glomerular filtration rate were associated with decreased risk of death (Table 2; Supplementary Table S3). The impact of timing on 90-day fatality was not clearly demonstrated (Supplementary Table S4).

**TABLE 2:** Risk factors for 28-days fatality after COVID-19 infection in KT recipients.

*N* = 878	Univariable HR (95%CI), *p* value	Multivariable HR (95%CI), *p* value
Age (×10 years-old)	1.49 (1.31–1.69), <0.001	**1.50 (1.32–1.70), <0.001**
Male gender	0.76 (0.57–1.00), 0.050	0.76 (0.58–1.00), 0.051
BMI (kg/m^2^)	1.01 (0.98–1.04), 0.443	**—**
Afro-Brazilian or mixed-race ethnicity	0.92 (0.69–1.22), 0.568	—
Living donor	0.83 (0.57–1.19), 0.307	—
Timer after KT (years)	1.01 (0.98–1.03), 0.627	—
Number of comorbidities		
None	REF	**REF**
1 or 2	1.27 (0.75–2.16), 0.370	1.34 (0.80–2.23), 0.260
≥3	1.81 (1.00–3.28), 0.050	**1.96 (1.10–3.48), 0.022**
IS regimen – ST	0.72 (0.42–1.25), 0.248	—
IS regimen – CNI	0.90 (0.49–1.65), 0.722	—
IS regimen – MPA/AZA	1.15 (0.63–2.08), 0.649	—
IS regimen – mTORi	0.44 (0.26–0.75), 0.003	**0.44 (0.27–0.72), 0.001**
ST pulse therapy ≤3 months	1.55 (0.68–3.57), 0.297	—
rATG ≤3 months	1.10 (0.39–3.05), 0.860	—
RAS blockade	1.22 (0.89–1.67), 0.209	—
Baseline eGFR (×10 ml/min/1.73 m^2^)	0.88 (0.82–0.94), <0.001	**0.87 (0.82–0.93), <0.001**
Quartiles of time after index case		
Q1: <72 days	REF	REF
Q2: 72–104 days	1.03 (0.72–1.48), 0.863	1.04 (0.73–1.48), 0.843
Q3: 105–140 days	0.75 (0.52–1.10), 0.145	0.80 (0.55–1.15), 0.228
Q4: >140 days	0.60 (0.40–0.90), 0.014	**0.65 (0.44–0.97), 0.037**

BMI, body mass index; KT, kidney transplant; IS, immunosuppressive; ST, steroid; MPA, mycophenolate; AZA, azathioprine; CNI, calcineurin inhibitor; mTORi, mammalian target of rapamycin inhibitor; rATG, rabbit anti-thymocyte globulin; RAS, renin-angiotensin system; eGFR, estimated glomerular filtration rate; HR, hazard ratio; CI, confidence interval; REF, reference.

Bold values denote statistical significance at the *p* < 0.05 level.

## Discussion

This national multicenter cohort suggests that COVID-19-associated fatality decreased over the first 6 months after the beginning of the pandemic. Changes in the demographic profile of infected patients, in the clinical presentation at diagnosis, and in pharmacological and non-pharmacological treatment options might explain this result.

The overall fatality rate was high and similar to that described in international published cohorts (15, 16, 18, 22). As a novelty, this cohort showed that the cumulative incidence of death within 28 days after diagnosis significantly decreased over time, and deaths occurred later. Changes in the demographic profile, mainly the reduction in the percentage of patients with multiple comorbid conditions, probably contributed to this finding, since the number of comorbidities was an independent risk factor for death (3). Despite the statistically significant trend for higher BMI over time, we believe that this finding is not clinically relevant. The reasons for the changes in the demographic profile over the months are not clear. The wide dissemination of the worst prognosis on the elderly, and patients with comorbidities might have resulted in intensification of protective measures in these individuals.

Other factors that might have impacted outcomes were the changes in the recommendations of the health care organizations, the higher availability of diagnostics tests, and the learning curve about disease diagnosis and management, leading to earlier and broader diagnosis, properly referred hospitalization, or better management of pharmacological and non-pharmacological interventions. In fact, the reduction in the percentage of patients with dyspnea, hypoxemia, and radiological chest findings suggest earlier demand for medical assistance, earlier clinical suspicion and diagnosis, and/or earlier hospitalization. The median time until intubation was prolonged by 3 days, suggesting improvements in the optimal use of non-invasive ventilation techniques. Unfortunately, we did not capture information about ventilatory management before invasive mechanical ventilation. Noteworthy, the interpretation of the downward trend in ICU admission must be cautious, since the availability of ICU beds is not uniform across the country’s centers and regions (2).

Interestingly, the improvement in the 90-day fatality was not evident. We believe that the 28-day mortality rate reflects disease severity, and prompt and proper diagnosis and treatment. In turn, 90-day mortality also seems to reflect intra-hospital care, such as preventing nosocomial infections, thromboembolic events, and other adverse events related to health care, malnutrition, and immobilization. Although these processes have probably also improved over the period, our study was not empowered to show this trend.

A clear change in the pharmacological supporting treatments was observed, which might also have impacted outcomes, mainly the higher use of high-dose steroids and anticoagulants (8, 9). The retrospective nature of a registry study, the absence of data on the onset of all interventions, and the diversity of COVID-19 management protocols in our continental country preclude any definitive conclusion about the efficacy of these strategies. We could not access information of patients who did not have access to medical care. The overwhelmed health system during the peaks of the pandemic could have hindered the arrival of more severe COVID-19 patients at the hospital, leading to deaths before hospitalization. In addition, despite the homogeneous number of patients in each quartile, groups have different duration, potentially hampering to capture the workload of periods with a higher incidence of cases and the effect of overwhelmed hospitals.

As another limitation, this study was limited to the first wave of the pandemic in Brazil, and reflected the pre-vaccination period. We do not have information on the viral genotype, which also might influence the clinical presentation and outcomes. However, at that time, the variants of concern leading to potential changes in the clinical profile and patients outcomes had not been identified yet (23). The imprecise definition of death cause in more than 10% of patients also impaired a better understanding of the reasons behind the reduction in fatality rates, as well as hampered the precise distinction between related and non-related COVID deaths.

It is also notable that a lower percentage of patients had their immunosuppressive regimen completely withdrawn over the study time. Despite plenty of *in vitro* studies suggesting the potential benefit of immunosuppressive drugs on the clinical outcomes of coronavirus infection (24–29), no clinical study supports robust conclusions. In the multivariable analysis, the use of mTOR inhibitors in the maintenance immunosuppressive regimen was associated with lower death risk. The reduction in SARS-CoV-2 replication after the inhibition of the Akt/mTOR/HIF-1 signaling pathway was previously demonstrated by a recently published *in vitro* study (29). However, no conclusion in this regard is feasible considering the limitation of the study design. Finally, despite the statistically significant linearly increasing trend through time, complex dynamics observed in some variables, such as the time between COVID-19 diagnosis and hospitalization, do not necessarily reflect clinically relevant changes.

Notwithstanding the above-mentioned limitations, inherent to registry data analysis, our study has important strengths: to the best of our knowledge, this is one of the largest multicenter national registers on COVID-19 in KT patients; the national representation is consistent with site activities and with COVID-19 incidence in the Brazilian States; a robust center-adjusted analysis was performed to minimize site-effect; and the selection of hospitalized patients only, excluding patients with mild COVID-19 forms, makes our sample more homogeneous as to the initial severity criterion.

In conclusion, this study suggests that the COVID-associated fatality in KT patients requiring hospitalization improved over the six first months of the pandemic. Prospective studies are of utmost needed to better understand the impact of each intervention on outcomes.

## Capsule Sentence Summary

This multicenter national Brazilian study accessed the fatality rates of COVID-19 among kidney transplanted patients over the first 6 months after the beginning of the pandemic. Using trend analysis, we could observe a decrease in the fatality rates from March to August 2020. A center-adjusted analysis was performed to explore the reasons for the improvement in the outcomes. Differences in demographics, clinical presentation, and treatment options might be involved in this trend.

## Data Availability

The raw data supporting the conclusions of this article will be made available by the authors, without undue reservation.

## The COVID-19-KT Brazil Study Group

Beyond the authors, the COVID-19-KT Brazil Study Group includes the following participants: Roger Kist^8^, Aline Lima Cunha Alcântara^2^, Maria Luiza de Mattos Brito Oliveira Sales^3^, Mario Abbud Filho^9^, Katia Cronenberge Sousa^10^, Roberto Ceratti Manfro^11^, Tomás Pereira Júnior^12^, Maria Eduarda Heinzen de Almeida Coelho^13^, Marilda Mazzali^21^, Marcos Vinicius de Sousa^21^, Juliana Bastos Campos^14^, Nicole Gomes Campos Rocha^15^, Tania Leme da Rocha Martinez^17^, Joao Egidio Romao Junior^17^, Maria Regina Teixeira Araújo^17^, Sibele Lessa Braga^17^, Marcos Alexandre Vieira^16^, Elen Almeida Romão^22^, Miguel Moysés Neto^22^, Juliana Aparecida Zanocco^23^, Auro Buffani Claudino^23^, Gustavo Guilherme Queiroz Arimatea^19^, Tereza Azevedo Matuck^20^, Alexandre Tortoza Bignelli^24^, Maria Ferneda Puerari^24^, José Hermógenes Rocco Suassuna^25^, Suzimar da Silveira Rioja^25^, Rafael Lage Madeira^26^, Sandra Simone Vilaça^26^, Carlos Alberto Chalabi Calazans^27^, Daniel Costa Chalabi Calazans^27^, Patricia Malafronte^28^, Luiz Antonio Miorin^28^, Larissa Guedes da Fonte Andrade^29^, Filipe Carrilho de Aguiar^29^, Fabiana Loss de Carvalho Contieri^30^, Karoline Sesiuk Martins^30^, Helady Sanders Pinheiro^31^, Emiliana Spadarotto Sertório^31^, André Barreto Pereira^32^, David Jose^9^; Barros Machado^33^, Carolina Maria Pozzi^34^, Leonardo Viliano Kroth^34^, Lauro Monteiro Vasconcellos Filho^36^, Rafael Fabio Maciel^37^, Amanda Maíra Damasceno Silva^38^, Ana Paula Maia Baptista^39^, Pedro Augusto Macedo de Souza^40^, Marcus Lasmar^41^, Luciana Tanajura Santamaria Saber^42^, Lilian Palma^43^.

^21^Hospital de Clínicas da Universidade de Campinas-UNICAMP, Campinas, SP, Brazil; ^22^Divisão de Nefrologia, Faculdade de Medicina de Ribeirão Preto da Universidade de São Paulo (FMRP-USP), Ribeirão Preto, SP, Brazil; ^23^Hospital Santa Marcelina, São Paulo, SP, Brazil; ^24^Hospital Universitário Cajuru, Curitiba, PR, Brazil; ^25^Hospital Universitário Pedro Ernesto, Rio de Janeiro, RJ, Brazil; ^26^Hospital Felício Rocho, Belo Horizonte, BH, Brazil; ^27^Hospital Marcio Cunha, Ipatinga, MG, Brazil; ^28^Santa Casa de Misericórdia de São Paulo, São Paulo, SP, Brazil; ^29^Hospital das Clínicas da UFPE Universidade Federal de Pernambuco, Recife, PE, Brazil; ^30^Hospital do Rocio, Campo Largo, PR, Brazil; ^31^Hospital Universitário da Universidade Federal de Juiz de Fora, Juiz de Fora, MG, Brazil; ^32^Hospital Marieta Konder Bornhausen, Itajai, SC, Brazil; ^33^Hospital Alemão Osvaldo Cruz, São Paulo, SP, Brazil; ^34^Hospital Evangélico, Curitiba, PR, Brazil; ^35^Hospital São Lucas da PUCRS, Porto Alegre, RS, Brazil; ^36^Hospital Meridional, Cariacica, ES, Brazil; ^37^Hospital Nossa Senhora das Neves, João Pessoa, PB, Brazil; ^38^Hospital Antonio Targino, Campina Grande, PB, Brazil; ^39^Hospital São Rafael, Salvador, BA, Brazil; ^40^Santa Casa de Misericórdia de Belo Horizonte, Belo Horizonte, MG, Brazil; ^41^Hospital Universitário Ciências Médicas, Belo Horizonte, MG, Brazil; ^42^Santa Casa de Misericórdia de Ribeirão Preto, Ribeirão Preto, SP, Brazil; ^43^Centro Médico de Campinas, Campinas, SP, Brazil.

## Supplementary Material

Supplementary Figure S1 Cumulative incidence of deaths of SARS-CoV-2-infected kidney transplant patients within 90 days.

Supplementary Table S1 Laboratory tests and chest radiological findings of kidney transplanted patients at COVID-19 diagnosis across quartiles of time.

Supplementary Table S2 Graft losses after COVID-19 diagnosis.

Supplementary Table S3 Risk factors for 28-days fatality after COVID-19 infection in KT recipients.

Supplementary Table S4 Risk factors for 90-days fatality after COVID-19 infection in KT recipients.

## Abbreviations

AKI, Acute kidney injury; AUC-ROC, Area Under the Receiver Operating Curve; COVID-19, Coronavirus disease 2019; eGFR, Glomerular filtration rate; ESKD, End-stage kidney disease; GLMM, Generalized Linear Mixed Models; IQR, Interquartile range; IRB, Institutional Review Board; KT, Kidney transplant; rATG, Antithymocyte globulin; RT-PCR, Reverse-transcription polymerase chain reaction; sCr, Serum creatinine; ST, Steroid; Δ sCr, Delta serum creatinine.
