# Supplementary material for: Temporal Reduction in COVID-19-Associated Fatality Among Kidney Transplant Recipients: The Brazilian COVID-19 Registry Cohort Study
Source: Transpl Int. 2022 Feb 1;35:10205. doi: 10.3389/ti.2022.10205 (PMC8842378; doi:10.3389/ti.2022.10205)

**Table S1. Laboratory tests and chest radiological findings of kidney transplanted patients at COVID-19 diagnosis across quartiles of time**

|  | **Non-**  **missing**  **cases** | **Total**  **N = 878** | **Q1**  **N = 227** | **Q2**  **N = 214** | **Q3**  **N = 219** | **Q4**  **N = 218** | **p*_for-trend_*** |
| --- | --- | --- | --- | --- | --- | --- | --- |
| **LABORATORY TESTS** |  |  |  |  |  |  |  |
| ***Lymphocytes (cells/mm^3^)*** | 632 | 715 (458-1,092) | 683 (440-1049) | 700 (434-1080) | 738 (504-1206) | 763 (502-1063) | 0.179 |
| ***Hemoglobin (g/dL)*** | 670 | 12.0 (10.4-13.5) | 11.8 (10.0-13.2) | 11.7 (10.3-13.4) | 12.1 (10.5-13.5) | 12.1 (10.513.6) | 0.115 |
| ***Platelets (cells/mm^3^)*** | 670 | 175,000 (133,000-230,000) | 178,000 (129,000-230,000) | 180,500 (137,000-234,500) | 171,000 (121,500-224,550) | 170,500 (134,000-230,250) | 0.709 |
| ***CRP (mg/dL)*** | 603 | 24 (7-96) | 17.6 (6.8-80.3) | 27.5 (7.3-112.5) | 32.4 (10.4-100.9) | 24.1 (5.8-79.2) | 0.582 |
| ***LDH (U/L)*** | 508 | 344 (237-466) | 352 (244-491) | 350 (232-481) | 346 (236-475) | 313 (235-425) | 0.032 |
| ***AST (U/L)*** | 562 | 31 (22-46) | 55 (33-84) | 51 (33-75) | 29 (22-44) | 29 (21-40) | **0.033** |
| ***ALT (U/L)*** | 570 | 24 (16-38) | 25 (16-38) | 25 (16-37) | 25 (15-45) | 21 (16-35) | 0.542 |
| ***CPK (U/L)*** | 311 | 82 (43-143) | 77 (39-154) | 75 (41-130) | 92 (44-160) | 86 (47-142) | 0.412 |
| ***Ferritin (mg/dL)*** | 305 | 963 (479-1,761) | 1,768 (482-3,516) | 1,048 (480-2,000) | 1,107 (469-1,755) | 916 (450-1,448) | 0.543 |
| ***Na^+^ (mEq/L)*** | 609 | 135 (132-138) | 135 (132-138) | 135 (131-138) | 135 (132-138) | 136 (133-139) | 0.125 |
| ***Δ SCr (mg/dL)*** | 681 | 0.4 (0.1-1.1) | 0.4 (0.0-0.9) | 0.5 (0.1-1.2) | 0.5 (0.1-1.2) | 0.3 (0.1-1.0) | 0.999 |
| **RADIOLOGICAL FINDINGS** | 726 |  |  |  |  |  |  |
| ***No abnormality*** |  | 27 (3.7) | 4 (2.1) | 4 (2.3) | 7 (3.8) | 12 (6.7) | **0.015** |
| ***GGO*** |  | 637 (87.7) | 167 (87.4) | 161 (93.1) | 162 (89.0) | 147 (81.7) | 0.057 |
| ***Consolidations*** |  | 314 (43.3) | 76 (39.8) | 75 (43.4) | 82 (45.1) | 81 (45.0) | 0.282 |

Legend: CRP: C-reactive protein; LDH: lactic dehydrogenase, AST: aspartate transaminase; ALT: alanine transaminase; CPK:creatine phosphokinase; Na+: serum sodium; Δ SCr: delta serum creatinine; GGO: ground-glass opacities

Trend analysis for categorical and continuous data were performed using Cochran–Armitage test and Jonckheere-Terpstra test, respectively.

**Table S2. Graft losses after COVID-19 diagnosis.**

| **n** | **Age (years-old)** | **Gender** | **Time after KT** | **IS regimen at COVID-19 diagnosis** | **eGFR at COVID-19 diagnosis (ml/min/1.73m^2^)** | **Changes in IS regimen**^§^ | **Graft loss time after COVID-19 diagnosis (days)** | **Attributable causes of Graft loss** |
| --- | --- | --- | --- | --- | --- | --- | --- | --- |
| #1 | 37 | M | 21.9 years | CsA-ST-MPA | 24.3 | No change | 9 | Immune IF/TA + AKI |
| #2 | 32 | F | 15.3 years | MPA-ST | 7.6* | MPA withdrawal | 11 | Nonimmune IF/TA + AKI |
| #3 | 52 | M | 2 months | TAC-ST-MPA | 23.8 | Complete withdrawal | 19 | Nonimmune IF/TA + AKI |
| #4 | 42 | F | 3 years | TAC-ST-MPA | 9.5* | Complete withdrawal | 45 | Nonimmune IF/TA + AKI |
| #5 | 76 | M | 9 months | TAC-ST-SRL | Not available | Complete withdrawal | 32 | Nonimmune IF/TA + AKI |
| #6 | 71 | M | 1 month | TAC-ST-SRL | 8.6* | Complete withdrawal | 20 | Nonimmune IF/TA + AKI |
| #7 | 45 | M | 4 months | TAC-ST-SRL | 10.4 | Complete withdrawal | 16 | Nonimmune IF/TA + AKI |
| #8 | 34 | F | 11 months | TAC-ST-MPA | 25.0 | Complete withdrawal | 69 | Nonimmune IF/TA + AKI |
| #9 | 51 | M | 1 month | TAC-ST-SRL | < 10^#^ | Complete withdrawal | 23 | Primary non-function |
| #10 | 23 | F | 1 days | No IS drug was introduced^&^ | < 10^#^ | Not applicable | 43 | AMR |
| #11 | 52 | F | 7 months | TAC-ST | 16.8 | Complete withdrawal | 72 | HIVAN + AKI |
| #12 | 38 | F | 8.2 years | TAC-ST-MPA | 25.6 | Complete withdrawal | 70 | Nonimmune IF/TA + AKI |
| #13 | 52 | F | 10.7 years | TAC-ST | 23.7 | Complete withdrawal | 22 | Immune IF/TA + AKI |
| #14 | 50 | M | 7.9 years | TAC-ST | 12.2 | No change | 34 | Nonimmune IF/TA + AKI |

Abbreviations: M: male; F: female; KT: kidney transplant; IS: immunosuppressive; CsA: cyclosporin; ST: steroid; MPA: mycophenolate; TAC: tacrolimus; SRL: sirolimus; eGFR: estimated glomerular filtration rate; IF/TA: interstitial fibrosis and tubular atrophy; AKI: acute kidney injury; AMR: antibody mediated rejection; HIVAN: HIV nephropathy.

*Patients with eGRF < 10 mL/min/1.73m^2^ on conservative treatment

^#^Patients on delayed graft function

^&^ Patient diagnosed with COVID-19 in the first 48h after KT

^§^ There is no information about the time between diagnosis and graft loss in which the IS change was performed

**Table S3. Risk factors for 28-days fatality after COVID-19 infection in KT recipients**

| N = 878 | **Univariable**  HR (95%CI), p value | **Multivariable**  HR (95%CI), p value |
| --- | --- | --- |
| Age (x 10 years-old) | 1.47 (1.29-1.67), <0.001 | **1.48 (1.31-1.68), <0.001** |
| Male gender | 0.77 (0.58-1.02), 0.071 | 0.77 (0.59-1.01), 0.063 |
| BMI (Kg/m^2^) | 1.01 (0.98-1.04), 0.384 | **-** |
| Afro-Brazilian or mixed-race ethnicity | 0.93 (0.70-1.23), 0.595 | - |
| Living donor | 0.82 (0.57-1.19), 0.301 | - |
| Timer after KT (years) | 1.01 (0.98-1.03), 0.656 | - |
| Number of comorbidities |  |  |
| *None* | REF | **REF** |
| *1 or 2* | 1.29 (0.76-2.20), 0.341 | 1.36 (0.82-2.27), 0.231 |
| *≥ 3* | 1.83 (1.01-3.33), 0.047 | **2.00 (1.12-3.55), 0.018** |
| IS regimen – ST | 0.72 (0.42-1.26), 0.249 | - |
| IS regimen – CNI | 0.92 (0.50-1.67), 0.774 | - |
| IS regimen – MPA/AZA | 1.14 (0.63-2.06), 0.665 | - |
| IS regimen – mTORi | 0.44 (0.26-0.77), 0.004 | **0.45 (0.27-0.73), 0.001** |
| ST pulse therapy ≤ 3 months | 1.39 (0.62-3.10), 0.417 | - |
| rATG ≤ 3 months | 1.14 (0.42-3.11), 0.791 | - |
| RAS blockade | 1.22 (0.89-1.67), 0.210 | - |
| Baseline eGFR (x 10 mL/min/1.73m^2^) | 0.88 (0.82-0.94), <0.001 | **0.87 (0.82-0.93), 0.001** |
| Time after the diagnosis of the index case (months) | 0.88 (0.80-0.97), 0.009 | **0.90 (0.82-0.99), 0.024** |

Legend: BMI: body mass index; KT: kidney transplant; IS: immunosuppressive; ST: steroid; MPA: mycophenolate; AZA: azathioprine; CNI: calcineurin inhibitor; mTORi: mammalian target of rapamycin inhibitor; rATG: rabbit anti-thymocyte globulin; RAS: renin-angiotensin system; eGFR: estimated glomerular filtration rate; HR:hazard ratio; CI: confidence interval; REF: reference

**Table S4. Risk factors for 90-days fatality after COVID-19 infection in KT recipients**

| N = 878 | **Univariable**  HR (95%CI), p value | **Multivariable**  HR (95%CI), p value |
| --- | --- | --- |
| Age (x 10 years-old) | 1.53 (1.38-1.70), <0.001 | **1.54 (1.39-1.71), <0.001** |
| Male gender | 0.98 (0.78-1.25), 0.893 | - |
| BMI (Kg/m^2^) | 1.02 (0.99-1.04), 0.126 | **-** |
| Afro-Brazilian or mixed-race ethnicity | 0.82 (0.65-1.04), 0.101 | - |
| Living donor | 0.81 (0.60-1.10), 0.181 | - |
| Timer after KT (years) | 1.02 (1.00-1.04), 0.060 | 1.01 (1.00-1.03), 0.164 |
| Number of comorbidities |  |  |
| *None* | REF | **REF** |
| *1 or 2* | 1.20 (0.79-1.83), 0.392 | 1.21 (0.81-1.82), 0.351 |
| *≥ 3* | 1.67 (1.03-2.70), 0.037 | **1.69 (1.06-2.69), 0.028** |
| IS regimen – ST | 0.96 (0.58-1.59), 0.881 | - |
| IS regimen – CNI | 0.98 (0.59-1.61), 0.928 | - |
| IS regimen – MPA/AZA | 1.09 (0.67-1.77), 0.737 | - |
| IS regimen – mTORi | 0.47 (0.30-0.74), 0.001 | **0.45 (0.30-0.66), <0.001** |
| ST pulse therapy ≤ 3 months | 1.81 (0.96-3.41), 0.066 | **1.61 (1.03-2.51), 0.035** |
| rATG ≤ 3 months | 0.89 (0.39-2.04), 0.790 | - |
| RAS blockade | 0.99 (0.76-1.29), 0.955 | - |
| Baseline eGFR (x 10 mL/min/1.73m^2^) | 0.90 (0.85-0.95), <0.001 | **0.89 (0.85-0.94), <0.001** |
| Quartiles of time after index case |  |  |
| *Q1: < 72 days* | REF | REF |
| *Q2: 72 - 104 days* | 1.06 (0.77-1.44), 0.728 | 1.08 (0.79-1.47), 0.625 |
| *Q3: 105 - 140 days* | 0.86 (0.63-1.18), 0.360 | 0.91 (0.67-1.25), 0.572 |
| *Q4: > 140 days* | 0.73 (0.53-1.02), 0.064 | 0.80 (0.58-1.11), 0.181 |

Legend: BMI: body mass index; KT: kidney transplant; IS: immunosuppressive; ST: steroid; MPA: mycophenolate; AZA: azathioprine; CNI: calcineurin inhibitor; mTORi: mammalian target of rapamycin inhibitor; rATG: rabbit anti-thymocyte globulin; RAS: renin-angiotensin system; eGFR: estimated glomerular filtration rate; HR: hazard ratio; CI: confidence interval; REF: reference


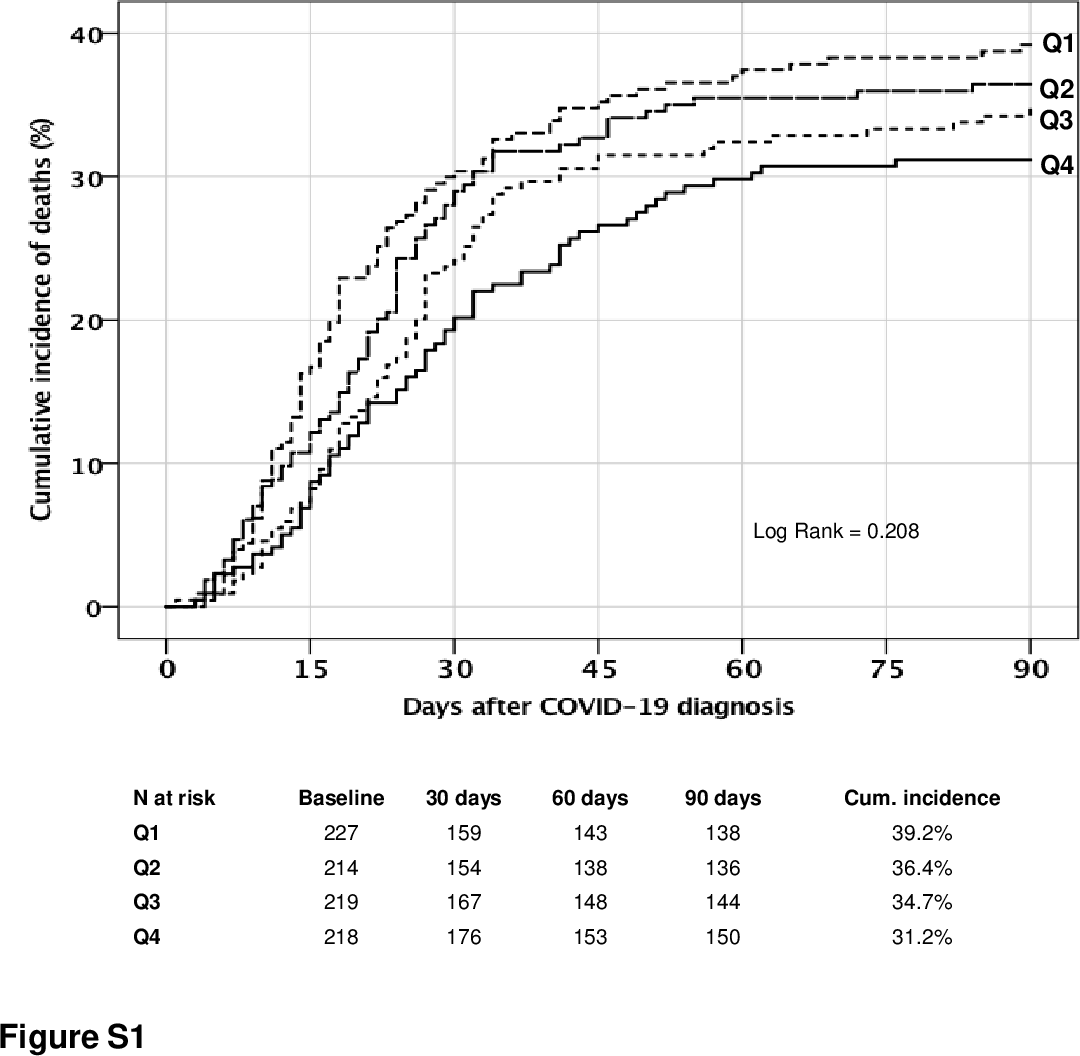

Supplement: Supplementary file 1 [file DataSheet1.docx]
